# The Mini-Cog, Clock Drawing Test, and Three-Item Recall Test: Rapid Cognitive Screening Tools with Comparable Performance in Detecting Mild NCD in Older Patients

**DOI:** 10.3390/geriatrics6030091

**Published:** 2021-09-16

**Authors:** Panita Limpawattana, Manchumad Manjavong

**Affiliations:** Division of Geriatric Medicine, Department of Internal Medicine, Faculty of Medicine, Khon Kaen University, Khon Kaen 40002, Thailand; lpanit@kku.ac.th

**Keywords:** Mini-Cog, clock drawing test, three-item recall test, mild cognitive impairment, MCI, mild neurocognitive disorder, mild NCD

## Abstract

Background: Early mild neurocognitive disorder (mild NCD) detection can allow for appropriate planning and delay disease progression. There have been few studies examining validated mild NCD detection tools. One such tool that may be of use is the Mini-Cog, which consists of the clock drawing test (CDT) and three-item recall. Methods: This study aimed to compare the diagnostic properties of the Mini-Cog, the CDT alone, and the three-item recall test alone in mild NCD detection according to DSM-5 criteria. The participants were older patients attending the medicine outpatient clinic. Area under receiver operating characteristic (ROC) curve (AUC) analysis was used to compare the tools’ accuracy. Results: A total of 150 patients were enrolled, 42 of whom were diagnosed as having mild NCD. The AUCs of ROC curves of the three-item recall, CDT, Mini-Cog1, and Mini-Cog2 were 0.71, 0.67, 0.73, and 0.71, respectively (*p* = 0.36). The sensitivity of the tools was 85.7%, 66.7%, 57.4%, and 69% respectively. The tests performed similarly in participants with ≤6 years of education (*p* = 0.27) and those with >6 years of education (*p* = 0.49). Conclusions: All tools exhibited similar acceptable performance in detecting mild NCD and were not affected by education. These convenient tools might be suitable for use in clinical practice.

## 1. Background

Major neurocognitive disorder (or dementia) is an important problem for the healthcare system, as it is incurable, causes suffering to patients and their families, and increases healthcare costs [1]. Early detection of dementia is critical in that it allows for the planning of appropriate management, early education of patients and families regarding disease prognosis, shared decision making for patient life planning, and improvements in patients’ quality of life. Mild neurocognitive disorder (mild NCD) is a stage of pre-dementia and must be recognized early to be treated effectively. While there is a high probability that this condition will progress to be dementia, some patients have experienced reversion to cognitive normality [2]. There are numerous reversible causes of dementia and various strategies that can be employed to delay the progression of cognitive impairment [3,4,5,6].

Previous studies have examined the performance of brief neuropsychological tools, such as the Montreal Cognitive Assessment (MoCA), Mini-Mental State Examination (MMSE), Rowland Universal Dementia Assessment Scale (RUDAS), and Informant Questionnaire on Cognitive Decline in the elderly (IQCODE), in detecting mild NCD [7,8,9,10,11,12]. Among these tools, the MoCA appears to yield the highest sensitivity and specificity for mild NCD screening and has been validated in various parts of the world [7,9,13]. However, the MoCA is time-consuming and has several important limitations. For example, the one-point correction for under 12 years of education might not be appropriate for older patients with lower levels of education in some regions, especially in rural areas [13]. In Thailand—which is poised to become a superaged society within the next 10 years—a majority of older adults have ≤4 years of education [14]. The MoCA might, thus, not be suitable for detecting mild NCD in this population. A validated test that is more accurate in detecting mild NCD in patients with low levels of education may be more suitable for use in this setting.

The Mini-Cog is one such neuropsychological test that has been effective in detecting patients with dementia [15,16,17]. Although its accuracy varies by region and method of interpretation, it has been shown to yield high sensitivity and specificity for detecting cognitive impairment [16,18,19]. The test, consisting of a clock drawing test (CDT) and three-item recall test, takes only 3 min to administer, making it suitable for use in a primary care setting. Moreover, a previous study found that administration of the CDT alone allowed for the differentiation of mild NCD patients from normal older adults with good sensitivity and excellent specificity [18]. Additionally, patients with mild NCD usually have episodic memory deficit [19], which means that a recall test on its own may also be useful in screening for mild NCD. A systematic review and meta-analysis evaluated the performance of recall tests in mild NCD diagnosis and found that they were more effective than the MMSE and MoCA, especially in amnestic mild NCD patients [20].

There have been few studies examining the diagnostic properties of the Mini-Cog, three-item recall test, and CDT for mild NCD detection in the Thai population. The primary aim of this study was, thus, to evaluate the performance these tests in mild NCD patients. The secondary aim was to evaluate the effect of education level on their efficacy.

## 2. Materials and Methods

### 2.1. Participants and Setting

**Figure 1:**
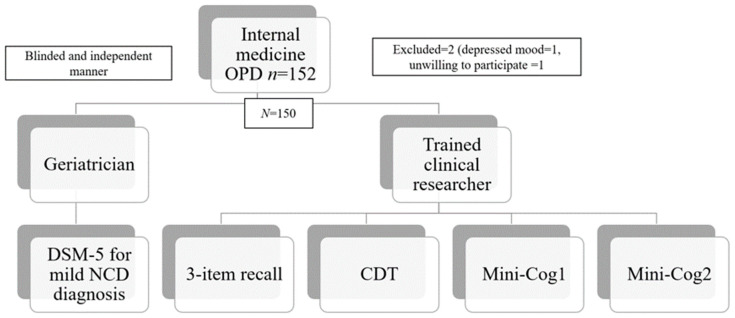
Study flow. Note: DSM-5, Diagnostic and Statistical Manual of Mental Disorders; mild NCD, mild neurocognitive disorder.

This study was part of a project entitled “The performance of the Rowland Universal Dementia Assessment Scale (RUDAS), recall test, and Mini-Cog in the screening of mild cognitive impairment” [21]. Data were collected between January 2020 and March 2021. Participants in this cross-sectional study were selected according to the following inclusion criteria: (1) age ≥60 years and (2) no apparent acute illness that could contribute to the performance of the tests such as infection, acute stroke, acute coronary syndrome, or delirium. Participants were visitors to the outpatient clinic of Srinagarind Hospital’s Department of Internal Medicine (Khon Kaen University). The exclusion criteria included a history of psychiatric disorder, major neurocognitive disorder (dementia), congenital mental retardation, mental illness, long-term use of antipsychotic drugs, and severe visual, hearing, or limb dysfunction. Patients with depression (defined as a score >9 on the Thai version of the Patient Health Questionaire-9 [PHQ-9]) [22], those with impaired instrumental activities of daily living (iADLs; defined as Chula ADL index <9) [23], those who were unable to communicate in Thai or the local language, and those who were reluctant to complete the tests were also excluded. Of the 152 patients who were eligible, one was excluded for depression, and another was unwilling to participate. The study flow is shown in Figure 1.

### 2.2. Operational Definition

#### Mild Neurocognitive Disorder (Mild NCD)

Mild neurocognitive disorder was defined according to the Diagnostic and Statistical Manual of Mental Disorders (DSM-5) criteria: (A) evidence of modest cognitive decline from a previous level of performance in one or more cognitive domains, (B) the cognitive deficits do not interfere with the capacity for independence in everyday activities, (C) the cognitive deficits do not occur exclusively in the context of delirium, and (D) the cognitive deficits are not better explained by another mental disorder [24].

### 2.3. Instrument

#### Mini-Cog1 and Mini-Cog2

**Figure 2:**
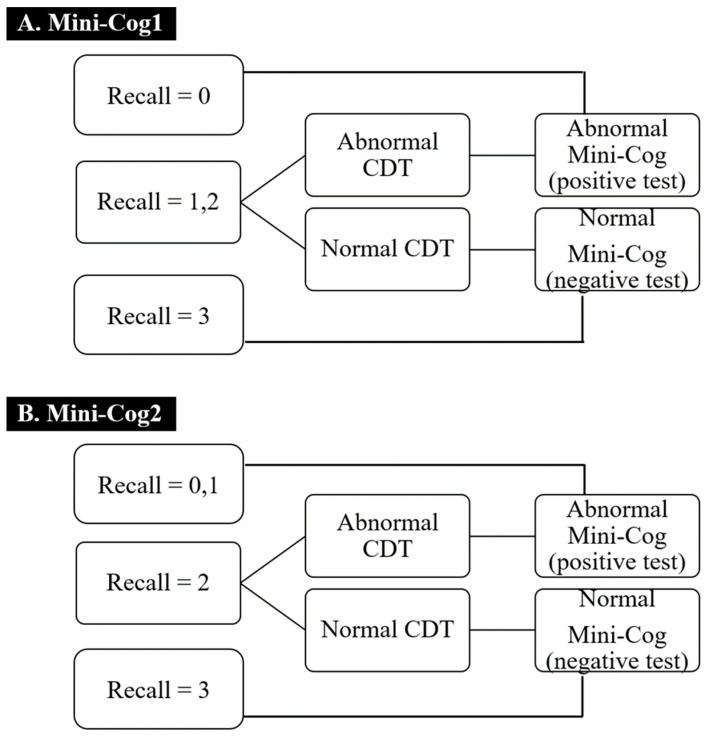
Scoring of the Thai version of the Mini-Cog1 (**A**) and Mini-Cog2 (**B**) [25].

As mentioned above, the Mini-Cog consists of a three-item recall test and a CDT. The three-item recall is scored as a maximum of three points, one for each word that is correctly recalled. In the CDT, the patient is asked to spontaneously draw a circular clock displaying a particular time (11:10). A three-point technique is used for scoring: one point for drawing a circle, one point for drawing the correct numbers on the clock, and one point for drawing the hands in the correct position. A score of 3 is considered normal. The Thai version of the Mini-Cog was evaluated according to the scoring rules described by Kusalaruk and Nakawiro (2012) [25], as shown in Figure 2.

### 2.4. Three-Item Recall Test

This study used the three items from the Thai version of the Mini-Cog to test short-term delayed recall. The score ranges from 0–3, with one point counted for each item correctly recalled [25].

### 2.5. Clock Drawing Test

In this study, the CDT was scored based on the Thai version of the Mini-Cog. Results were either “normal” or “abnormal” [25]. A three-point technique was used for scoring: one point for drawing a circle, one point for drawing the correct numbers on the clock, and one point for drawing the hands in the correct position. A score of 3 is considered normal.

### 2.6. Procedure

This study was approved by the Khon Kaen University Faculty of Medicine Ethics Committee for Human Research in accordance with the Helsinki Declaration. All participants provided written informed consent prior to data collection. Demographic data of participants, including age, sex, years of education, marital status, and comorbid diseases, were collected using convenience sampling. Subsequently, a trained clinical researcher administered the three-item recall test, CDT, and Mini-Cog (scoring as Mini-Cog1 and Mini-Cog2. Then, a geriatrician determined the participant’s mild NCD diagnosis according to DSM-5 criteria. The researcher and the geriatrician were each blinded to the other’s results.

### 2.7. Statistical Analysis

Descriptive statistics were used for the demographic and clinical characteristics of the participants and were presented as percentage, mean, and standard deviation (SD). If the distribution of the data was not normal, median and interquartile range were used instead. Area under ROC curve analysis was used to compare the overall accuracy of each of the tests for detecting mild NCD. The classification of the AUC ROC score was as follows: AUC = 0.5 was considered no discrimination, 0.6 ≥ AUC > 0.5 was considered poor discrimination, 0.7 ≥ AUC > 0.6 was considered acceptable discrimination, 0.9 ≥ AUC > 0.7 was considered excellent discrimination, and AUC > 0.9 was considered outstanding discrimination [26]. Additionally, an exploratory data analysis of test performance by education level was performed. All data analysis was performed using STATA v10.0 (StataCorp, College Station, TX, USA).

## 3. Results

### 3.1. Participant Characteristics

Demographic and clinical data are presented in Table 1. Of the 150 patients tested, 42 were determined to have mild NCD (28%). The majority of patients with mild NCD were women, and they were more likely to be older and have diabetes mellitus than those in the intact cognition group. The mild NCD group had lower item recall scores and abnormal CDT, Mini-Cog1, and Mini-Cog2 scores.

### 3.2. Mild NCD Screening Accuracy of the Three-Item Recall Test, CDT, Mini-Cog1, and Mini-Cog2

The sensitivity and specificity of the tools are shown in Table 2. The three-item recall test produced the highest sensitivity compared to the others. Moreover, the three-item recall test exhibited the highest and the CDT exhibited the lowest overall performance in screening for mild NCD, but this difference was not statistically significant (*p* = 0.36; see Table 2 for the AUCs of ROC curves). Educational level did not affect the performance of any of the screening tools (Table 3).

## 4. Discussion

The Mini-Cog is a brief and convenient neuropsychological test. However, most studies have been conducted in patients with dementia and not those with mild NCD [25,27]. Mild NCD patients can be either amnestic or non-amnestic. Amnestic mild NCD commonly presents as memory deficit and usually progresses to Alzheimer’s disease, whereas non-amnestic mild NCD usually presents as impairment of executive function, attention, and visuospatial skills and usually develops into other types of dementia [28,29,30,31]. We found the overall performances of the Mini-Cog (both Mini-Cog1 and Mini-Cog2), three-item recall test, and CDT in differentiating mild NCD patients from normal older adults to be acceptable according to the AUCs of the ROC curve. These tools measure different cognitive domains (memory and executive function) affected by both types of mild NCD.

Although various methods have been used to interpret the Mini-Cog, such as overall score (0–3 points for the three-item recall plus 0–2 points for the CDT, with a total score of 0–2 points suggesting a high risk of dementia) [32], the two-step method used for the Mini-Cog1 is common [19,33,34]. One study from Thailand found that the Mini-Cog2 had better diagnostic properties for detecting dementia (72.8% sensitivity and 97.6% specificity) than the Mini-Cog1 (66.7% sensitivity and 98.4% specificity) [25]. Our study supports the results of the previous one, whereby Mini-Cog2 showed the better sensitivity to detect mild NCD than Mini-Cog1 (Table 2). This could be explained by zero or one point in the recall test in the Mini-Cog2 being interpreted to be cognitive impairment regardless of CDT, whereas one point in the recall test in the Mini-Cog1 needed the result of CDT to be the final interpretation. However, we found the overall performance of the two tests to be comparable, suggesting that either could be used for mild NCD screening.

The overall performance of the CDT and three-item recall in this study was comparable to that of the Mini-Cog1 and Mini-Cog2. While the three-item recall performed slightly better than the CDT, this difference was not statistically significant. A tool with high sensitivity might be the most appropriate for mild NCD detection. Our findings showed that three-item recall appeared to be the best option (Table 2). This could be explained by the fact that the three-item recall test is effective in detecting the memory impairment characteristic of amnestic mild NCD, which is more common than the non-amnestic type [28,30]. Our results were similar to those of several previous studies [20,33]. For example, a systematic review and meta-analysis of 108 diagnostic studies found that recall tests performed better than the CDT in terms of diagnostic accuracy in mild NCD detection (sensitivity 89% vs. 58% and AUC 0.94 vs. 0.7) [20]. Another study from Germany compared the Mini-Cog and CDT for dementia detection and concluded that the Mini-Cog had higher discriminatory power than the CDT (sensitivity of 86.8% and 73.1%, respectively) [33].

Few studies have evaluated the effect of education on the Mini-Cog’s performance, and the results of previous studies have been inconsistent. A study in a Brazilian population, for example, concluded that Mini-Cog was not effective in participants with <5 years of education [34]. Another study showed that the Mini-Cog was more useful than the MMSE (sensitivity 89.2% vs. 73.7%) in participants with low levels of education (≤8 years of education) [33]. Similarity, a study in Chinese outpatients found that the Mini-Cog had higher sensitivity than the MMSE to detect mild NCD in patients with no education (87.5% vs. 56.25%), ≤6 years of education (86.49% vs. 64.86%), and >6 years of education (84.62% vs. 56.45%) [35]. According to the results of our study, we found that the Mini-Cog1, Mini-Cog2, three-item recall, and CDT were not affected by level of education, suggesting that these are appropriate screening tools in undereducated populations.

There were several limitations to this study. Firstly, the results might lack generalizability, since the study was conducted in a tertiary care hospital where patients had greater severity of disease and greater risk of mild NCD, resulting in a higher prevalence of mild NCD than in a general setting. Secondly, diagnosis was mainly based on clinical judgement without laboratory tests. Thirdly, the study design could have resulted in misclassification bias. Fourthly, only three participants had no formal education; hence, the results may not reflect the performance of these tools in that population. Lastly, the sample size was quite small and derived from a single study. Further studies should be performed to evaluate the utility of these tools among patients with no formal education and the performance of the Mini-Cog in combination with other neuropsychological tests to detect mild NCD.

## 5. Conclusions

The Mini-Cog1, Mini-Cog2, three-item recall test, and CDT exhibited similarly acceptable diagnostic properties for detecting mild NCD and were not affected by the patient’s level of education. All are quick, easy, and convenient to administer and should be used as alternative tools in clinical practice for mild NCD detection among Thai older adults, especially the three-item recall test which exhibited the best sensitivity and overall performance compared to the others.

## Figures and Tables

**Table 1:** Demographic and clinical characteristics of the participants.

Variables	Non-Mild NCD *N* = 108 (72%)		Mild NCD *N* = 42 (28%)	
Age, median (IQR1,3)	67	(62.5, 73.5)	72	(66, 75)
Women, *n* (%)	53	(49.1)	25	(59.5%)
Years of education, *n* (%)				
0	0	(0)	3	(7.1)
≤6 years	34	(31.5)	25	(59.5)
6 to 12 years	28	(25.9)	7	(16.7)
>12 years	46	(42.6)	7	(16.7)
Marital status, *n* (%)				
Single	3	(2.8)	2	(4.8)
Married	82	(75.9)	26	(61.9)
Divorced	8	(7.4)	1	(2.4)
Widow	15	(13.9)	13	(30.9)
Comorbid disease(s) *, *n* (%)				
DM	43	(39.8)	23	(54.8)
HT	81	(75)	33	(78.6)
DLD	72	(66.6)	15	(35.7)
CKD	27	(25)	4	(9.5)
AF	7	(6.5)	4	(9.5)
IHD	2	(1.9)	2	(4.8)
CVA	5	(4.6)	4	(9.5)
OSA	7	(6.5)	2	(4.8)
Number of items recall, median (IQR1,3)	3	(2, 3)	2	(1, 2)
Abnormal CDT, *n* (%)	36	(33.3)	28	(66.7)
Positive Mini-Cog1, *n* (%)	20	(18.5)	27	(65.3)
Positive Mini-Cog2, *n* (%)	29	(26.9)	29	(69.1)

* DM: diabetes mellitus, HT: hypertension, DLD: dyslipidemia, CKD: chronic kidney disease, AF: atrial fibrillation, IHD: ischemic heart disease, CVA: cerebrovascular accident, OSA: obstructive sleep apnea.

**Table 2:** Comparison of the overall performance of the three-item recall test, CDT, Mini-Cog1, and Mini-Cog2.

Tests	Sensitivity	Specificity	AUC of ROC Curve	95%CI	*p*-Value
3-item recall	85.7%	56.5%	0.71	0.63–0.78	*p* = 0.36
CDT	66.7%	66.7%	0.67	0.58–0.75	
Mini-Cog1	57.4%	85.4%	0.73	0.65–0.81	
Mini-Cog2	69%	73.1%	0.71	0.63–0.79	

**Table 3:** Comparison of the overall performance of the three-item recall test, CDT, Mini-Cog1, and Mini-Cog2 by education level.

Tests	AUC (95%CI)	
	≤6 Years *N* = 62 *	>6 Years *N* = 88 **
3-item recall	0.71 (0.60–0.82)	0.71 (0.60,0.82)
CDT	0.57 (0.46–0.68)	0.59 (0.45,0.73)
Mini-Cog1	0.68 (0.57–0.80)	0.67 (0.53–0.81)
Mini-Cog2	0.69 (0.57–0.80)	0.65 (0.51–0.79)

* *p* = 0.27, ** *p* = 0.49.

## Data Availability

No new data were created or analyzed in this study. Data sharing is not applicable to this article.
